# Effect of peripheral blood lymphocyte count on the efficacy of immunotherapy combined with TKI in the treatment of advanced liver cancer

**DOI:** 10.3389/fimmu.2024.1467429

**Published:** 2024-10-24

**Authors:** Qian Zhao, Lei Wang, Huilan Fu, Yuqin Zhang, Qiankun Xie

**Affiliations:** ^1^ Department of Infectious Diseases, Nanfang Hospital, Southern Medical University, Guangzhou, China; ^2^ Department of Imaging Center, Nanfang Hospital, Southern Medical University, Guangzhou, China; ^3^ Department of Gastroenterology, Guangzhou Development District Hospital, Guangzhou, China; ^4^ Department of Radiation Oncology, Nanfang Hospital, Southern Medical University, Guangzhou, China

**Keywords:** hepatocellular carcinoma, TKI, PD1, peripheral blood lymphocyte count, combination therapy

## Abstract

**Background and aims:**

Compared with tyrosine kinase inhibitor (TKI) monotherapy, TKI combined with PD1 can improve the therapeutic effect of liver cancer and has been widely used in clinical practice. However, there is a lack of effective biomarkers to identify patients who would benefit more from this combination therapy. Therefore, this study aimed to evaluate whether baseline lymphocyte counts can identify patients with liver cancer who would benefit from targeted immune combination therapy.

**Methods:**

Data from patients with hepatocellular carcinoma (HCC) who received TKIs or TKIs in combination with PD1 between June 2018 and June 2020 were retrospectively collected. The patients were divided into high and low groups based on the median absolute count of peripheral lymphocytes before systemic therapy and differences in overall survival (OS) and progression-free survival (PFS) between TKI and TKI+PD1 were compared between the two groups.

**Results:**

In total, 72 patients were included in this study, with a median follow-up of 1.5 years. Both PFS and OS in the TKI+PD1 group showed a good prognostic trend (p = 0.058 and p = 0.077, respectively). Subgroup analyses based on peripheral blood lymphocyte counts showed that the combination regimen had a significant PFS and OS advantage only in patients with high peripheral blood lymphocyte counts (p = 0.036 and p = 0.031, respectively), but not in patients with low absolute peripheral blood lymphocyte counts (p = 0.819 and p = 0.913, respectively).

**Conclusions:**

Peripheral blood lymphocyte count is a simple and effective biomarker that can be used to identify patients with liver cancer who will benefit more from TKI+PD-1 combination therapy.

## Introduction

In recent years, new therapies such as targeted therapy with sorafenib/lenvatinib, and immunotherapy with PD-1/PD-L1 inhibitors have become the treatment of choice for liver cancer (1–3). Tumor vascular abnormalities lead to hypoxia and acidosis in the tumor microenvironment, which causes immunosuppression through a variety of mechanisms, and anti-angiogenic can normalize the blood vessels around the tumor and improve the microenvironment, thereby promoting the effect of immunotherapy (4, 5). Compared with the lower response rate of monotherapy, combination immunotherapy based on TKIs has shown promising efficacy in advanced liver cancer (6–9). For example, the Keynote524 studies showed that the objective response rate to lenvatinib in combination with PD1 reached 46% (8). However, many patients do not benefit from the combination regimen, which causes adverse reactions, such as hepatitis/pneumonia caused by immunotherapy, seriously reducing the patients’ quality of life of patients and affecting subsequent antitumor therapy (1). Phase III clinical trial results showed that in the lenvatinib plus pembrolizumab group, 71 (18%) of 395 patients discontinued any study treatment because of treatment-related adverse events versus 42 (11%) of 395 patients in the lenvatinib plus placebo group. The treatment-related grade 3–4 adverse events were also higher in the combination group than alone lenvatinib (243 [62%] vs 224 [57%]) (7).

Considering the toxicity and increased treatment costs of the combination regimen, identifying which patients are more suitable for the two-drug combination is a clinically meaningful direction; however, current research in this area is very limited. Lymphopenia is associated with poor prognosis in multiple cancer types and can be used to predict the efficacy of tumor immunotherapy (10–12). Therefore, in this study, we aimed to investigate whether baseline lymphocyte counts could predict the probability of benefits from targeted immune combination therapy in patients with liver cancer.

## Materials and methods

### Patients

This retrospective study was conducted at Nanfang Hospital, Southern Medical University, and was approved by the Ethics Committee of Nanfang Hospital, Southern Medical University. We retrospectively analyzed patients with continuous liver cancer who received TKIs alone or TKIs in combination with PD1 at our hospital between June 2018 and June 2020. We included patients who met the following criteria: 1) age > 18 years; 2) diagnosis of liver cancer by clinical or pathological examination; 3) adequate recording of baseline blood routine tests; 4) PS score 0–2 points; and 5) the first systemic therapy was targeted therapy with lenvatinib or sorafenib, alone or in combination with PD-1 antibody. Patients were excluded if they had 1) history of organ transplantation, 2) immunodeficiency diseases, 3) incomplete medical data or loss to follow-up, 4) prior treatment with other systems, and 5) were administered immune checkpoints for second-and multiline therapy.

### Systemic treatment

All patients provided written informed consent before undergoing systemic therapy. Sorafenib orally 400 mg 1/day. Lenvatinib 8 mg orally or at 12 mg 1/day. PD-1 inhibitor alone, camrelizumab (200 mg), toripalimab (240 mg), sintilimab(200 mg) or pembrolizumab(200 mg)once every 3 weeks as an intravenous infusion or nivolumab (3 mg/kg every 2 weeks). The reduction or discontinuation of treatment was determined by the clinician, depending on the disease status and adverse effects.

### Data collection

Patient baseline characteristics, such as age, sex, ECOG Score and AFP, were obtained from their electronic medical records. Hematological parameters for all patients were concentrated in the 1 week before the first systemic therapy. Evaluation of patient efficacy was based on imaging information using the mRESIST criterion, and patient survival information was collected via telephonic follow-up. OS was defined as the time from the first administration of systemic therapy to the patient’s death or loss to follow-up. PFS was defined as the time from the first administration of systemic therapy to tumor progression.

### Statistical analysis

Categorical or continuous variables were compared between groups using the chi-square test or t-test. Kaplan–Meier analysis was used for OS and PFS, and the log-rank test was used for comparisons between groups. P < 0.05 was considered statistically significant. All statistical analyses were performed using the SPSS software.

## Results

### Patient characteristics

In total, 72 patients were included in this study; their baseline characteristics are shown in **Table 1**. Most patients had hepatitis B virus (HBV) infection, BCLC stage C, Eastern Cooperative Oncology Group (ECOG) performance status of 0–1, and Child–Pugh class A. A total of 36 (50.0%) patients received concomitant local therapy, including TACE/HAIC/radiotherapy/radiofrequency ablation. Among the 72 patients, 29 received targeted immune combination therapy as first-line treatment, whereas 43 received targeted therapy alone. A higher proportion of patients in the TKI+PD-1 group received lenvatinib than those in the TKI group (37.9% vs. 14.0%, p = 0.019).

**Table 1:** Baseline characteristics of the patients.

Variables	All patients (n = 70)	TKI alone	TKI+PD1	P value
Age (yrs)	52.9 ± 12.1	54.2 ± 11.2	51.0 ± 13.4	0.283
Gender				
Male	66 (91.7%)	40 (93.0%)	26 (89.7%)	0.612
Female	6 (8.3%)	3 (7.0%)	3 (10.3%)	
Pathogeny				
HBV-related	69 (97.8%)	41 (95.3%)	28 (96.6%)	0.802
Others	3 (2.2%)	2 (4.7%)	1 (3.4%)	
ECOG Score				
0-1	49 (68.1%)	28 (65.1%)	21 (72.4%)	0.137
>1	23 (31.9%)	23 (34.9%)	8 (27.6%)	
Child-Pugh class				
A	45 (62.5%)	26 (60.5%)	19 (65.5%)	0.664
B	27 (37.5%)	17 (39.5%)	10 (34.5%)	
AFP (ng/mL)				
< 200	22 (30.6%)	14 (32.6%)	8 (27.6%)	0.653
≥ 200	50 (69.4%)	29 (67.4%)	21 (72.4%)	
BCLC stage				
B	3 (2.1%)	2 (4.7%)	1 (3.4%)	0.802
C	69 (97.8%)	41 (95.3%)	28 (96.6%)	
Types of TKIs				
Sorafanib	48 (66.7%)	37 (86.0%)	18 (62.1%)	0.019
Lenvatinib	24 (33.3%)	6 (14.0%)	11 (37.9%)	
MVT				
Yes	54 (75.0%)	32 (74.4%)	22 (75.9%)	0.89
No	18 (25.0%)	11 (25.6%)	7 (24.1%)	
EM				
Yes	38 (52.8%)	22 (51.2%)	16 (55.2%)	0.738
No	34 (47.2%)	21 (48.8%)	13 (44.8%)	
Local therapy				
Yes	36 (50.0%)	23 (53.5%)	13 (44.8%)	0.471
No	36 (50.0%)	20 (46.5%)	16 (55.2%)	

AFP, alpha-fetoprotein; BCLC, Barcelona Clinic Liver Cancer; ECOG PS, Eastern Cooperative Oncology Group performance status; EM, extrahepatic metastases; HBV, hepatitis B virus; MVT, macrovascular tumor thrombosis; TKIs, tyrosine kinase inhibitors.

### Overall survival and progression-free survival

**Figure 1:**
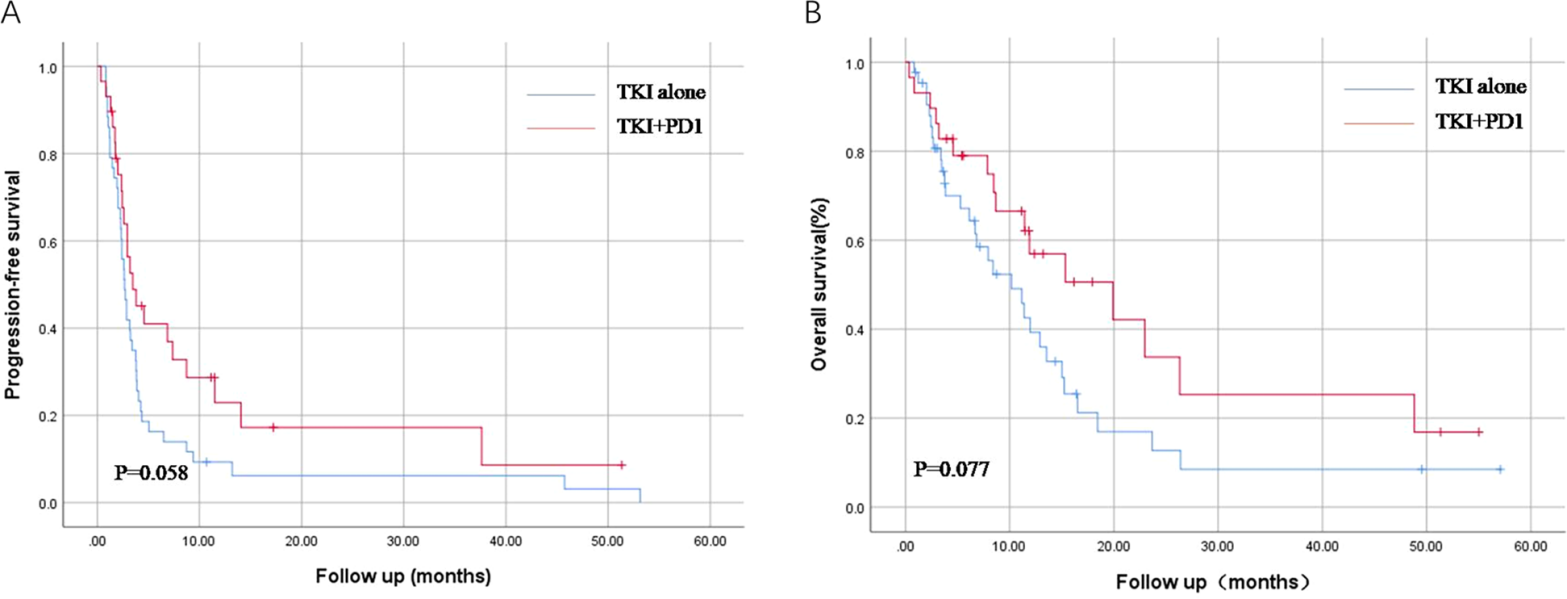
Kaplan–Meier curves for progression-free survival **(A)** and overall survival **(B)** in TKI alone and TKI+PD-1 group. TKIs, tyrosine Kinase Inhibitors. All statistical tests were two-sided.

The median follow-up period was 1.5 years. By the end of follow-up time, 46 of the 72 patients had a death outcome and 64 had disease progression. Compared to the TKI treatment group, the PFS and OS of the TKI+PD1 group showed a better prognostic trend. The median PFS (mPFS) was longer in the TKI+PD1 group (3.5 months, 95%CI 1.5–5.5) than in the TKI group (2.7 months, 95%CI 2.2–3.2) [p = 0.058; **Figure 1A**]. The median OS was 10.2 months, (95%CI 5.7–14.6) in the TKI group and 19.9 months, (95%CI 7.3–32.5) in the TKI+PD1 group [p = 0.077; **Figure 1B**].

### Survival analysis by absolute peripheral blood lymphocyte count

**Figure 2:**
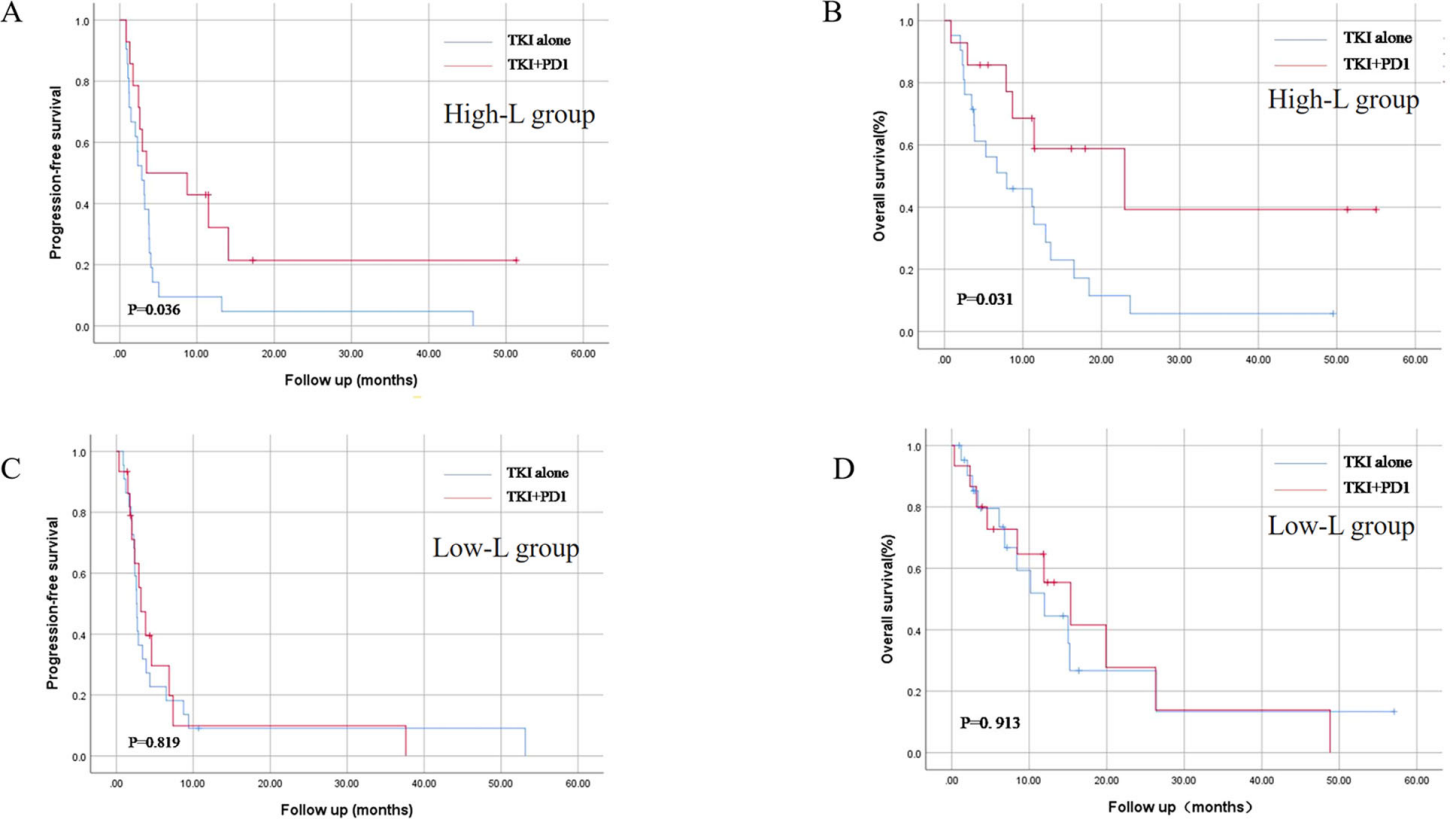
Kaplan–Meier curves for progression-free survival **(A, C)** and overall survival **(B, D)** in TKI alone and TKI+PD-1 group after stratification by peripheral blood lymphocyte count. TKIs, tyrosine Kinase Inhibitors. All statistical tests were two-sided.

Stratified analysis was performed based on the absolute peripheral blood lymphocyte count before systemic therapy. Patients were divided into high- and low-L groups based on the median absolute count of peripheral blood lymphocytes. In the high-L group, patients in the TKI+PD1 group showed longer mPFS compared with those who received TKIs [3.5 months, (95%CI 0.1–14.1) versus 2.9 months (95%CI 1.6–4.2), p = 0.036], and mOS [22.9 months, (95%CI 1.4–44.5) versus 7.9 months, (95%CI 0.1–16.0) p = 0.031; **Figures 2A, B**].

No significant difference was found in mPFS [2.6 months, (95%CI 2.3–3.0) versus 3.2 months (95%CI 1.7–4.7), p = 0.819], and mOS [11.9 months, (95%CI 5.9–18.1) versus 15.3 months (95%CI 7.6–23.1), p = 0.913; **Figures 2C, D**] between TKI and TKI+PD1 use in the low-L group.

Moreover, we also used the lower limit of the normal value of lymphocytes to distinguish between people with high and low lymphocytes, and we found the same phenomenon (**Additional File 1**: **Supplementary Figure S1**).

## Discussion

To date, there are no effective biomarkers to screen patients with cancer to identify those who are more suitable for TKI+PD1 rather than single-agent TKI use. In this study, we assessed whether peripheral blood lymphocyte count could be used as a prognostic marker for the combination regimen and found that patients with low lymphocyte counts did not receive additional benefit from the combination regimen compared with single-agent targeting. Thus, our results suggest that peripheral blood lymphocyte count can be used as a biomarker to identify patients with liver cancer who will benefit from TKI+PD-1 combination therapy.

The treatment of advanced liver cancer remains challenging, with molecularly targeted therapies such as sorafenib and lenvatinib having low response rates. Consequently, combination immunotherapy such as PD-1/PD-L1 monoclonal antibodies has become a trend in the treatment of liver cancer (1). Target-immune therapy has a higher objective response rate than single-agent targeting (6–9). Similarly, our data showed that targeted combined immunization can prolong overall patient survival, which supports the advantages of combination therapy. However, a significant proportion of patients do not benefit from additional combination therapy, and the overall toxicity of combination regimens is high. These issues have prompted clinicians to make granular treatment decisions.

Our study found that TKI+PD1did not improve the prognosis of patients with peripheral blood lymphocytopenia. Compared with the difficulty and heterogeneity of tissue biopsy, peripheral blood lymphocytes are a simple clinical test index, based only on a simple routine blood test. This can help roughly determine which patients do not require targeted immunotherapy drugs, especially considering the toxicity and cost of combination therapy.

Nonetheless, the mechanism of peripheral blood lymphopenia in targeted immune combination therapy remains unclear. It is speculated that the mechanism may be related to the key role of lymphocytes in tumor immunity. A low peripheral blood lymphocyte count suggests a preexisting immunosuppressive state, resulting in an inadequate tumor immune response (12–14).

In contrast, patients with advanced liver cancer in the context of hepatitis B often have cirrhosis, which contributes to the development of hypersplenism, which often manifests as a decrease in the number of peripheral blood cells, including peripheral blood lymphocytes (15). The results of our association analysis showed that patients with low peripheral blood lymphocytes are often accompanied by a decrease in platelets and leukocytes. Some studies have suggested that the cellular immune function of patients with hypersplenism is severely impaired (15, 16), which may be a possible reason why peripheral lymphocytes can predict target-immune combination therapy; further experiments are needed to verify this.

Our study had the following limitations. First, this was a single-center retrospective study and the small sample size limited further subgroup analyses. Moreover, patient data was mainly based on electronic medical records and telephone follow-up, and patients who were lost to follow-up may experience a certain degree of bias. Second, the study included a combination of systemic regimens, including lenvatinib and sorafenib, which may differ in prognostic outcomes depending on the choice of the drug. In addition, some patients received concomitant local therapies. These local treatments may have a certain impact on the interpretation of the results. Based on your suggestions, we further analyzed the situation of local treatments received during the same period in different groups and found that there was no statistical difference between the subgroups in whether or not local treatment was received, which weakened the impact of this factor to a certain extent (**Additional File 2**: **Supplementary Table S1**).

In conclusion, our study revealed that peripheral blood lymphocyte count is an objective and simple indicator to identify which patients with advanced HCC should receive TKI+PD1 as a first-line systemic therapy rather than TKI alone. Properly designed prospective studies are needed to further explore these interesting findings.

## Data Availability

The raw data supporting the conclusions of this article will be made available by the authors, without undue reservation.
