# Supplementary material for: Effect of peripheral blood lymphocyte count on the efficacy of immunotherapy combined with TKI in the treatment of advanced liver cancer
Source: Front Immunol. 2024 Oct 24;15:1467429. doi: 10.3389/fimmu.2024.1467429 (PMC11540664; doi:10.3389/fimmu.2024.1467429)
Supplement: Supplementary file 1 [file DataSheet1.docx]

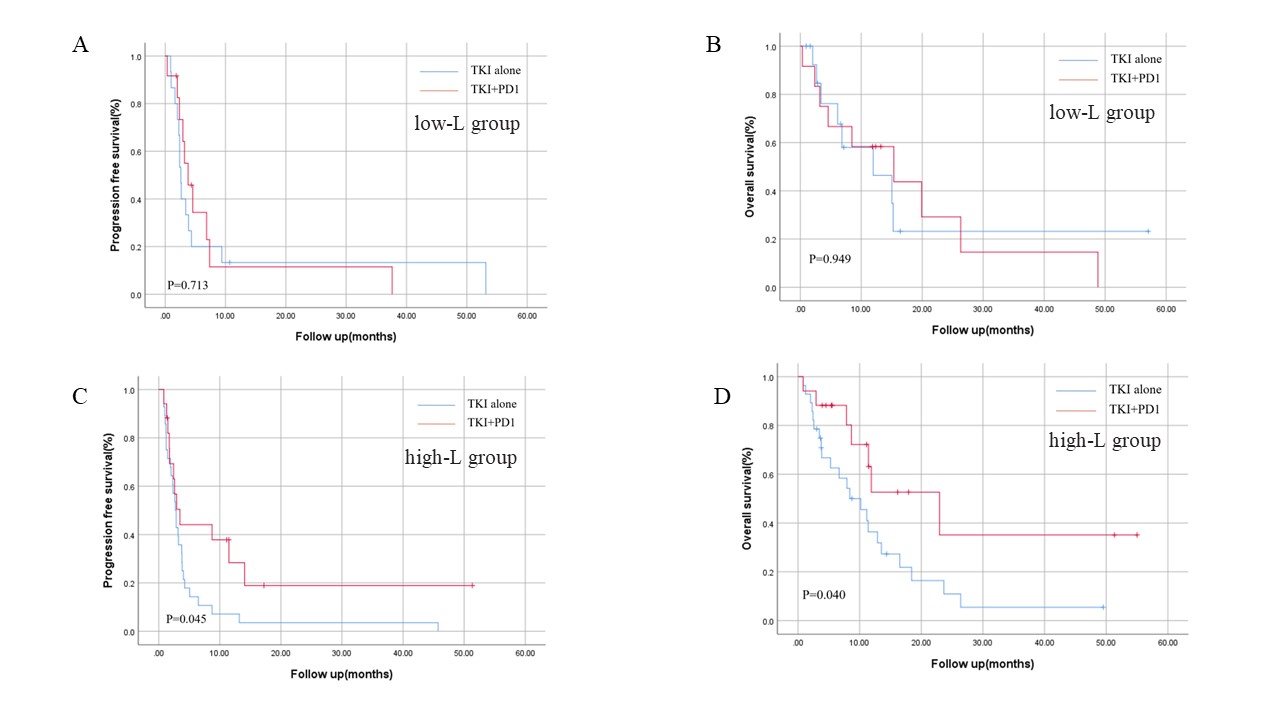


Figure S1. Kaplan–Meier curves for progression-free survival (a, c) and overall survival (b, d) in TKI alone and TKI+PD-1 group after stratification by the lower limit of the normal value of lymphocytes. TKIs, tyrosine Kinase Inhibitors. All statistical tests were two-sided.
