# Supplementary material for: Effect of peripheral blood lymphocyte count on the efficacy of immunotherapy combined with TKI in the treatment of advanced liver cancer
Source: Front Immunol. 2024 Oct 24;15:1467429. doi: 10.3389/fimmu.2024.1467429 (PMC11540664; doi:10.3389/fimmu.2024.1467429)
Supplement: Supplementary file 2 [file Table1.docx]

Table S1. Distribution of local treatments among subgroups

| Variables | All patients (n = 70) | low-L group | high-L group | P value |
| --- | --- | --- | --- | --- |
| Local therapy |  |  |  |  |
| Yes | 36（50.0%） | 17（45.9%） | 19（54.3%） | 0.479 |
| No | 36（50.0%） | 20（54.1%） | 16（45.7%） |  |

TKIs, tyrosine kinase inhibitors.
